# Factors influencing terminal cancer patients’ autonomous DNR decision: a longitudinal statutory document and clinical database study

**DOI:** 10.1186/s12904-022-01037-9

**Published:** 2022-08-27

**Authors:** Ru-Yih Chen, Ying-Chun Li, Kuang-Chieh Hsueh, Fu-Wei Wang, Hong-Jhe Chen, Tzu-Ya Huang

**Affiliations:** 1 Department of Family Medicine, Kaohsiung Veterans General Hospital, Kaohsiung, Taiwan, ROC; 2 Department of Business Management, Institute of Health Care Management, National Sun Yat-Sen University, No. 70. Lianhai Rd, Kaohsiung, Taiwan, ROC

**Keywords:** Cancer, Do-not-resuscitate orders, Family, Medical ethics, Personal autonomy, Terminal care

## Abstract

**Objective:**

Much of our knowledge of patient autonomy of DNR (do-not-resuscitate) is derived from the cross-sectional questionnaire surveys. Using signatures on statutory documents and medical records, we analyzed longitudinal data to understand the fact of terminal cancer patients’ autonomous DNR decision-making in Taiwan.

**Methods:**

Using the medical information system database of one public medical center in Taiwan, we identified hospitalized cancer patients who died between Jan. 2017 and Dec. 2018, collected their demographic and clinical course data and records of their statutory DNR document types, letter of intent (DNR-LOI) signed by the patient personally and the consent form signed by their close relatives.

**Results:**

We identified 1,338 signed DNR documents, 754 (56.35%) being DNR-LOI. Many patients had the first DNR order within their last week of life (40.81%). Signing the DNR-LOI was positively associated with being under the care of a family medicine physician prior to death at last hospitalization and having hospice palliative care and negatively associated with patient age ≥ 65 years, no formal education, having ≥ 3 children, having the first DNR order to death ≤ 29 days, and the last admission in an intensive care unit.

**Conclusions:**

A substantial proportion of terminal cancer patients did not sign DNR documents by themselves. It indicates they may not know their actual terminal conditions and lose the last chance to grasp time to express their life values and wishes. Medical staff involving cancer patient care may need further education on the legal and ethical issues revolving around patient autonomy and training on communicating end-of-life options with the patients. We suggest proactively discussing DNR decision issues with terminal cancer patients no later than when their estimated survival is close to 1 month.

## Background

There is an emphasis on equality in the relationship between medical care practitioners and their patients in modern medical care [1]. Physicians are expected to share medical decisions with their patients today. Therefore, in addition to providing medical care, physicians are expected to provide medical information and knowledge to help the patients and their family members come to suitable medical decisions. In addition to discussing the pros, cons, and feasibility of multiple options with patients, physicians are expected to empathetically and sensitively communicate in ways that reduce anxiety and conflict between patients and their family members, as well as show regard for the choices of their patients, since respect for patient autonomy is a revered contemporary bioethics principle [2].

Many countries have legislated that terminally ill patients can autonomously refuse CPR in favor of natural death [3, 4]. In Taiwan, the Physicians Law and Medical Care Law stipulated that medical personnel should administer cardiopulmonary resuscitation (CPR) to all dying patients without unreasonable delay [5, 6]. That is to say, medical staff must resuscitate every dying patient following the law regardless of the disease status, the individual’s life values, and autonomous will. However, it has been well-documented that the administration of CPR to terminal cancer patients usually only supports the maintenance of short-lived vital signs, does not effectively prolong life, and can worsen patients’ end-of-life quality [7–11].

In the Year 2000, the government in Taiwan passed the “Hospice and Palliative Care Act”, making it the first in Asia to recognize that end-stage patients have the right to choose a natural death [12]. This act stipulates that terminally ill patients can refuse CPR if they or their medical surrogates have signed the legal do-not-resuscitate letter of intent (DNR-LOI) documents. Every competent adult has the great right to personal end-of-life decisions. Signing the DNR-LOI is considered an exercise of an individual’s extraordinary refusal right to regular life-sustaining treatment. It must be done prudently when a person is in full control of his or her cognitive faculties and makes the voluntary decision in the presence of two witnesses. This change indicates the improvement of respect for personal medical autonomy because terminal dying resuscitation has no longer been a paternal authority policy.

The DNR-LOI can also be noted on the person’s national health insurance (NHI) card. The note has the same legal effect as that of the paper document. Only when a terminally ill patient has become unconscious or failed to express clear personal will and has not signed the DNR-LOI his/her close relative can sign a do-not-resuscitate consent form (DNR-CF). For those who do not have close relatives, a DNR order for the best interest of terminally ill patients can be issued after the examination of the hospice palliative care team.

Studies investigating medical autonomy issues usually administered questionnaires to explore knowledge, attitudes, and probable behaviors of patients, their family members, or medical workers. To the best of our knowledge, no study used longitudinal statutory DNR document data to investigate terminal cancer patients’ autonomy issues. Therefore, in an effort to obtain more objective data, we used collected data, including signed DNR documents, from the medical records of cancer patients who died hospitalized at one public medical center in southern Taiwan. The physician must confirm that the DNR document has been signed before issuing a DNR medical order. Physicians violating the law shall be subject to a fine, as well as suspension of practice or revocation of practice license, making the DNR document an excellent indicator. This study assumed that patients who had signed DNR-LOI had been informed of the terminality of their illness and their end-of-life care options by their attending physicians. Thus, a signed DNR-LOI can represent that the patient's medical autonomy has been respected. We further studied what variables would be associated with regard or disregard.

## Materials and method

### Data sources

In November 2019, we used one tertiary public medical center’s medical information system to identify hospitalized patients aged 20 years old and above with cancer diagnosis who died between 1 Jan 2017 and 31 Dec 2018 because the legal age for full capacity is 20 years old in Taiwan. We collected data related to the patients’ care six months prior to their death, including demographics, DNR document types and time of the first physician-issued DNR order, family meeting, last admission in hospice care or assigned ward and specialty of attending physician, admission dates, and status at discharge.

### Deceased cancer patient identification

Patients were identified as having a cancer diagnosis based on discharge records listing a primary disease classification ICD-10 code C00-C97. Status at discharge was determined by a code indicating “death” or “impending death discharge” on the medical record. The DNR code was derived from a medical order issued by the patient’s attending physician.

### DNR document types

A legal DNR document, LOI (signed by the patient) or CF (signed by a close relative), was identified based on a physician’s order supported by either paper documents, scanned files in electronic hospital records, or NHI card records. The DNR documents of each patient may contain both DNR-LOI and DNR-CF. Any patient with one DNR-LOI document was regarded as the DNR-LOI group.

### Physician specialties

Although DNR decisions may involve the participation of physicians with different specialties, our database did not include the details of all those involved. Therefore, we only used the specialty of the attending physician at last admission in our analysis, assuming that this physician would have the last opportunity to advocate for the patient’s autonomy.

### Hospice palliative care

In 2011, Taiwan’s National Health Insurance began to cover hospice palliative care services, including homecare, shared-care [13], and hospice ward admission. Any patient with one of these codes in the database was regarded as receiving hospice palliative care regardless of where they received this care.

### Statistical analysis

Hospital data were imported in Office Excel 2013 form into STATA version 14.0 for sorting and analysis. Data were first summarized descriptively and then analyzed by Chi-square test (χ2 test), One-Way ANOVA, and multivariate logistic regression. Collinearity diagnostics were performed to determine whether there were severe problems with multicollinearity for regression analysis. All p-values less than 0.05 were considered significant.

## Results

### Patient characteristics

**Fig. 1:**
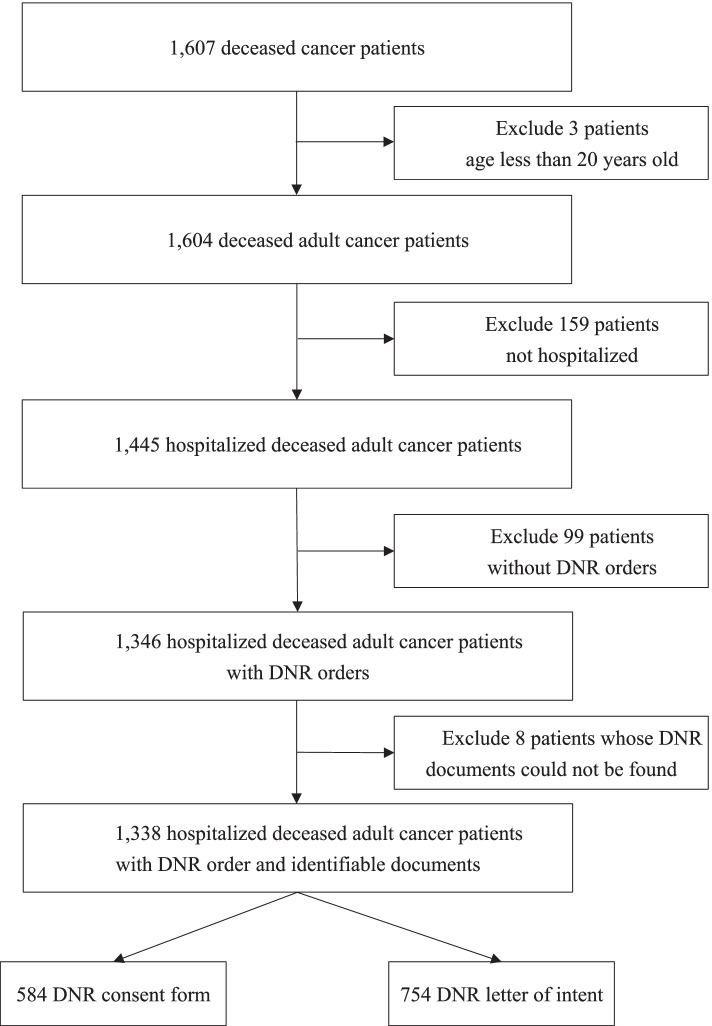
Flow chart of do-not-resuscitate (DNR) document identification

One thousand six hundred and seven cancer patients this medical center had treated died between 1 Jan 2017 and 31 Dec 2018. As can be seen in Fig. 1, a flowchart showing inclusion and exclusion of subjects, 1,445 of these patients had been hospitalized. Most of these hospitalized deceased cancer patients (1,346, 93.15%) had DNR orders. The DNR rate is similar to the average of hospitals enrolled in Taiwan’s Cancer Care Quality Assurance Measures accreditation program, which is 91.96 ± 0.64% for 2016–2020. We then excluded those for whom we could not find documents supporting physician orders and located the statutory DNR documents for 1,338 patients, 754 DNR-LOI (56.35%) and 584 DNR-CF (43.65%). Of the 754 DNR-LOI orders, 646 were supported with scanned files or paper documents (85.68%), 106 (14.06%) with a note on the NHI card, and two by documents signed (0.26%) by patient-appointed medical surrogate agents.

As can be seen in Table 1, most of the patients with identifiable DNR documents were male (60.61%), were aged ≥ 65 years (55.23%), had spouses (68.09%), had ≥ 3 children (51.27%), had academic education from elementary school to high school graduation (67.12%), and had received hospice palliative care (76.08%). A large proportion (40.81%) of them had the first DNR order within one week prior to death. Only 28.77% of these patients had a physician-conducted family meeting to disclose their terminal conditions.

**Table 1:** Characteristics of 1,338 hospitalized deceased adult cancer patients with DNR^a^ orders and identifiable documents

Variables (mean ± SD^b^)	Number (%)	Variables	Number (%)
Year		DNR document type	
2017	665 (49.70)	Letter of intent	754 (56.35)
2018	673 (50.30)	Consent form	584 (43.65)
Sex		Family meeting	
Male	811 (60.61)	No	953 (71.23)
Female	527 (40.39)	Yes	385 (28.77)
Age group (66.79 ± 14.20 years)		Hospice palliative care	
20–64	599 (44.77)	No	320 (23.92)
≥ 65	739 (55.23)	Yes	1018 (76.08)
Highest education level		Last admitted ward	
None	206 (15.40)	Intensive care unit	78 (05.83)
Primary to high school	898 (67.12)	Ordinary	693 (51.79)
Diploma or above	234 (17.49)	Hospice	567 (42.38)
Children number ( 2.59 ± 1.47)		Last admission service specialty	
0	134 (10.01)	Surgery	217 (16.22)
1–2	518 (38.71)	Internal medicine	402 (30.04)
≥ 3	686 (51.27)	Family medicine	719 (53.74)
Marital status		Final discharge status	
Married	911 (68.09)	Death	1059 (79.15)
Others^c^	427 (31.91)	Impending death	279 (20.85)
First DNR order to death length (31.70 ± 55.23 days)		Last hospital stay length (18.77 ± 19.21 days)	
0–7	546 (40.81)	0–7	433 (32.36)
8–29	428 (31.99)	8–29	640 (47.83)
≥ 30	364 (27.20)	≥ 30	265 (19.81)

^a ^DNR Do not resuscitate

^b ^SD Standard deviation

^c ^Including single, divorced, or widowed

### Factors influencing DNR document type

Table 2 summarizes the results of our comparison analysis between DNR-LOI and DNR-CF groups. Table 3 shows our multivariate logistic regression analyses for DNR-LOI. The regression analysis revealed the following to be positively associated with the signing of a DNR-LOI: having a family medicine practitioner as attending physician on last admission service (Odds ratio [OR] 2.714, confidence interval [CI] 1.659–4.438) and receiving hospice palliative care (OR 1.771, CI 1.257–2.497). The following variables were negatively associated with the signing of the DNR-LOI: being ≥ 65 years old (OR 0.592 CI 0.460–0.763), having no formal education (OR 0.574, CI 0.374–0.880), having ≥ 3 children (OR 0.575 CI 0.370–0.894), the first DNR order issued ≤ 7 days prior to death (OR 0.397, CI 0.283–0.556) and 8–29 days prior to death (OR 0.677, CI 0.491–0.933), and the last admission to an intensive care unit (ICU) (OR 0.347 CI 0.191–0.630).

**Table 2:** Analysis of demographic and clinical characteristics for different DNR^a^ document types

	DNR document type		*p*		DNR document type		*p*
	CF^b^ (%)	LOI^c^ (%)			CF (%)	LOI (%)	
Sex			0.908	Family meeting			0.084
Female	229 (39.21)	298 (39.52)		No	430 (73.63)	523 (69.36)	
Male	355 (60.79)	456 (60.48)		Yes	154 (26.37)	231 (30.64)	
Age group (Years)			< 0.001^*^	Hospice palliative care			< 0.001^*^
20–64	227 (38.87)	372 (49.34)		No	219 (37.50)	101 (13.40)	
≥ 65	357 (61.13)	382 (50.66)		Yes	365 (62.50)	653 (86.60)	
Highest education level			< 0.001^*^	Last admitted ward			< 0.001^*^
No formal education	120 (20.55)	86 (11.41)		Intensive care unit	62 (10.62)	16 ( 2.12)	
Primary to high school	375 (64.21)	523 (69.36)		Ordinary	350 (59.93)	343 (45.49)	
Diploma or above	89 (15.24)	145 (19.23)		Hospice	172 (29.45)	395 (52.39)	
Children number			< 0.001^*^	Last admission service specialty			< 0.001^*^
0	43 ( 7.36)	91 (12.07)		Surgery	124 (21.23)	93 (12.33)	
1–2	199 (34.08)	319 (42.31)		Internal medicine	247 (42.30)	155 (20.56)	
≥ 3	342 (58.56)	344 (45.62)		Family medicine	213 (36.47)	506 (67.11)	
Marital status			0.433	Final discharge status			0.002^*^
Married	193 (33.05)	234 (31.03)		Death	439 (75.17)	620 (82.23)	
Others	391 (66.95)	520 (68.97)		Impending death	145 (24.83)	134 (17.77)	
First DNR order to death length (days)			< 0.001^*^	Last hospital stay length (days)			0.810
≤ 7	326 (55.82)	220 (29.18)		0–7	184 (31.51)	249 (33.02)	
8–29	162 (27.74)	266 (35.28)		8–29	281 (48.11)	359 (47.62)	
≥ 30	96 (16.44)	268 (35.54)		≥ 30	119 (20.38)	146 (19.36)	

^a ^DNR Do-not-resuscitate

^b ^CF Consent form

^c ^LOI Letter of intent

^* ^*p*-value < 0.05

**Table 3:** Factors influencing signing of DNR^a^ letter of intent

Variables	Multivariate Analysis		p
	OR^b^	95% CI^c^	
Age group (Years)			
20–64	Reference		
≥ 65	0.592	0.460–0.763	< 0.001^*^
Highest education level			
No formal education	0.574	0.374–0.880	0.011^*^
Primary to high school	0.998	0.717–1.389	0.991
Diploma or above	Reference		
Children number			
0	Reference		
1–2	0.810	0.522–1.258	0.349
≥ 3	0.575	0.370–0.894	0.014^*^
First DNR order to death length (days)			
≤ 7	0.397	0.283–0.556	< 0.001^*^
8–29	0.677	0.491–0.933	0.017*
≥ 30	Reference		
Hospice palliative care			
No	Reference		
Yes	1.771	1.257–2.497	0.001^*^
Last admitted ward			
Ordinary	Reference		
Hospice	0.666	0.437–1.014	0.058
Intensive care unit	0.347	0.191–0.630	0.001^*^
Final discharge status			
Death	Reference		
Impending Death	0.945	0.702–1.273	0.710
Last admission service specialty			
Surgery	Reference		
Internal medicine	1.082	0.750–1.559	0.674
Family medicine	2.714	1.659–4.438	< 0.001^*^

^a^ DNR Do not resuscitate

^b^ OR Odds ratio

^c^ CI Confidence interval

Multivariate binomial logistic regression analysis. ^*^p-value < 0.05

Collinearity diagnostics: variance inflation factor = 2.08

## Discussion

DNR decision is an important end-of-life decision. It is considered important that patients be informed of the terminal nature of their disease, their end-of-life care options, and be given the opportunity to make these decisions by themselves [14]. In this study, we found that a large proportion (43.65%) of cancer patients who had been hospitalized did not sign these DNR documents by themselves. And many of them had the first DNR order within their last week of life (40.81%). Patients whose final attending physicians were family medicine practitioners, and those who had received hospice palliative care were more likely to have signed DNR documents in person. Patients who were older than 65 years, those with no formal education, those with ≥ 3 children, those who had the first DNR order prior to death ≤ 29 days, and those whose last admission was to ICU were less likely to have a personally signed DNR document.

### Increased DNR-LOI proportion in Taiwan

The signing of DNR documents conforms to the public’s perception that a terminal cancer patient should have the right to decide how he or she wishes to die [15]. Accepting DNR decisions made by terminal patients themselves shows regard for their autonomy. In this study, the proportion of personally signed DNR-LOI was more than half (56.35%), a significant increase compared to the 22% in another Taiwan study in 2013 [16]. This increase may be due to Taiwan’s NHI decision to pay for hospice palliative care provision and add the optional note of personal DNR wish on NHI cards promoted via hospital subsidies for community education [17]. It may also be due to the addition of respect for the autonomy of terminal patients to the criteria for hospital accreditation by the Ministry of Health and Welfare and an increase in the number of much older patients in Taiwan’s aging society that the people can gradually face the natural death process. In addition, the rise in the number of older patients who have received formal education in their early years would make them better able to understand their conditions and communicate with medical staff by themselves.

### Factors facilitating signing DNR-LOI

This study found that patients who were cared for by family medicine physicians prior to death at last hospitalization and those who had received hospice palliative care were more likely to have signed DNR-LOI. The Taiwan Family Medicine Association’s certification standards for specialist physicians’ training programs required that palliative care skills be well-trained in symptom control, communication, and medical ethics. Considering that all the family medicine practitioners have received this training, they would probably be more comfortable discussing end-of-life care options with terminal cancer patients and assisting them in signing the DNR documents in person than other medical specialists, such as surgeons and oncologists who may not be required to receive as much communication training and who may have more concern with other matters, including medical treatment and the prolongation of life for cancer patients [18–20]. According to the Taiwan Hospice Palliative Medicine Society statistics, 771 (70.04%) of their members had family medicine backgrounds in 2020. It indicates that family medicine specialists have considerable enthusiasm for providing terminal care.

Taiwan’s NHI covers the expense of hospice palliative care provided by a professionally trained team composed of doctors, nurses, and social workers. Following NHI regulations, the team members must have 80 h of hospice palliative care training and have at least 20 h of continuing education each year. The course includes ethics and law of terminal care, communication skills, and specialist consultation. Hence, patients who had received hospice palliative care were also more likely to have signed DNR-LOI. And hospice palliative care teams may pay more attention to patient autonomy while serving their patients.

Previous studies have found that most terminal patients want to be informed of their actual condition, especially by physicians [21–23]. Therefore, physicians must learn skills to help them relay bad news, express empathy, give supportive feedback, and accomplish consensus for end-of-life care. The commonly used clinical models for these included SPIKES (setting, perception, invitation or information, knowledge, empathy, and summarized or strategize) six steps as well as the Japanese SHARE (supportive environment, how to deliver bad news, additional information, and reassurance and emotional support) model, and the shared decision-making model [24–26].

### Factors hindering signing DNR-LOI

We found that advanced age, having more than three children, having no formal education, delay of the first DNR order, and being cared for in an ICU negatively affected the patient’s likelihood of signing a DNR-LOI. Studies have found that, in addition to disadvantages in cancer treatment options, age-related physical decline, delay in tumor detection, psychosocial factors, low chances of seeking medical attention, and high comorbidities may all hinder the process of truth-telling for older patients [27, 28]. However, it has also been found that older patients desire to understand the cancer diagnosis and prognosis as much as younger ones [23, 29, 30].

Aged people are often disadvantaged in accessing healthcare resources due to poor health literacy, deteriorating performance status, lower education levels, and economic dependence [31]. We also found signing the DNR-LOI was negatively associated with patients aged ≥ 65 years in the present study. Taiwan has been an aged society since 2018. Some aged terminal patients may lose the opportunity to have personal DNR decisions because of underlying cognitive dysfunctions or sudden physical deterioration due to multiple co-morbidities. The public is still not used to openly discussing end-of-life issues in geriatric care for worry of offending the elderly’s taboos. Traditionally, medical staff is more accustomed to prioritizing young family members to discuss end-of-life care plans for aged patients in Taiwan. Many young people have never personally dealt with matters related to the death of their elders. They may take avoidance measures when facing the issues of end-of-life treatments of their beloved elders, that is, not telling the truth and postponing decisions. Aged patients may be unable to know the fact that they have been in terminal status when they can still master autonomy. Nevertheless, the Hospice and Palliative Care Act’s legislation indicates that most citizens have agreed with the futility of life-prolonging treatment for dying terminal cancer patients. The aged patients’ close relatives usually signed the DNR-CF to forgo the elderly from CPR suffering in the final. So, the total DNR rate signed by close relatives remains high.

Culturally speaking, in Asia, the topic of death is traditionally avoided. For example, many public buildings, including hospitals, have no “4^th^” floor because the word “four” is a near homonym of the word “death” in many East Asia countries [32]. Having three or more children negatively affects the likelihood of DNR-LOI. Studies have found that family members often try to interfere with the physician’s communication with the patient of his or her cancer diagnosis and prognosis in Asia, UK, US, and Canada [21, 33–37]. This reluctance, combined with the East-Asian value of harmony and stability among family members, some of whom may not have come to terms with the thought of impending death, make end-of-life care discussion hard [38]. The more family members involved, the more difficult it is to reach a consensus. Studies found that family meetings for end-of-life care decisions, especially when conducted prophylactically or proactively, effectively improve family and staff satisfaction and reduce resource utilization [36, 39]. Advanced care planning education can also increase people’s knowledge and positive attitudes towards death [40]. We suggested that such programs be carried out regularly through media promotion and community activities to increase social acceptance.

Close families who sign the DNR-CF usually worry that the patients will suffer emotional breakdowns, cranky thoughts, or even lose the will to live if they learn the terminal fact. Medical staff should empathize with their grief and anxiety about the upcoming loss of loved ones. It is essential to proactively provide a channel for the family to express emotions and help them deal with the patient’s characteristics and cultural attributes. Medical staff can remind family members that they know the patient most. They may facilitate patients to make medical decisions according to personal life values in a way patients can understand and accept. Such as, the word “worst health condition” may be used instead of “death” or “tube and mechanical treatment” for “resuscitation” when discussing DNR issues.

We also found that a lack of formal education, delayed DNR discussion, and the last admission to ICU reduced the likelihood that DNR-LOI would be signed. Medical staff and relatives may worry about the ability of poorly educated patients to understand the physiological and medical details regarding their terminal cancer conditions and the content of the DNR-LOI. In addition, these patients are mostly much older people with hearing, visual impairment, and other comorbidities that might interfere with their decision-making. The timing DNR discussion is also relevant. Those with the first DNR orders at ≤ 29 days from death usually have more weakened physical conditions, have more stressful physical symptoms, and be less cognitively able to deal with technical and sensitive discussions. Physicians at this point would be less likely to initiate the discussion. Similarly, the severe medical conditions, coupled with respiration machines, nasogastric tubes, and sedative agents, make this discussion difficult in the ICU. We recommend that medical staff initiate the conversation about DNR issues with terminal cancer patients personally no later than when their estimated survival is close to 1 month.

### Strengths and limitations

The strength of our study lies mainly in the longitudinal statutory DNR document and clinical data used in its analysis. While most patient autonomy studies are cross-sectional and questionnaire-based, our analysis used two years’ clinical data and objective legal document records maintained in quite representative tertiary medical centers in Taiwan. The results explicitly present the fact of autonomous DNR decisions by terminal cancer patients in clinical care. The limitation of our study is that the kind of data extracted from medical discharge records does not contain clinical details. For example, hepatic coma, sepsis, and disturbance of consciousness caused by brain metastases may prevent patients from mastering autonomy. And the electrical medical records do not contain their attitudes towards death, which may be related to the timing of autonomous DNR decision-making.

## Conclusion

Based on the statutory medical documents and clinical data, we found factors facilitating and hindering terminal cancer patients from making their personal DNR decisions. The literature review also found similar situations in different cultural regions. It can be implied from these findings that more attention should be paid to earlier DNR issue discussions with terminal cancer patients. We suggest the timing should be no later than when their estimated survival approaches one month. Medical staff involving cancer patient care may need further education on patient autonomy and training on communicating end-of-life options. And we suggest further studies to investigate the outcomes of various communication training models for early end-of-life issues discussion or advance care planning based on clinical data.

## Data Availability

The datasets used and analyzed during the current study are available from the corresponding author on reasonable request.

## Abbreviations

CPR: Cardiopulmonary resuscitation
DNR-LOI: Do-not-resuscitate letter of intent
DNR-CF: Do-not-resuscitate consent form
NHI: National health insurance
